# Indications for breast magnetic resonance imaging in Brazil: past, present, and future

**DOI:** 10.1590/0100-3984.2021.0056

**Published:** 2021

**Authors:** Almir Galvão Vieira Bitencourt, Rubens Chojniak

**Affiliations:** 1 Department of Imaging - A.C.Camargo Cancer Center, São Paulo, SP, Brazil.

*Dear Editor,*

In the previous issue of **Radiologia Brasileira**, Ferreira et al.^(1)^ described the main indications for breast magnetic resonance imaging (MRI) examination at a referral center for the diagnosis and treatment of breast cancer in Brazil. The results presented by those authors corroborate the findings of a previous study conducted by our group, carried out at another referral center in Brazil and published in the journal in 2011^(2)^. Although they were performed at facilities in different states and in different time periods (2014-2018 and 2008-2009, respectively), both studies showed that most breast MRI examinations performed in Brazil are requested for the evaluation of patients in whom the mammography and ultrasound findings are inconclusive. Although some studies have shown the benefit of MRI in some specific situations within that context^(3,4)^, that indication has little foundation in the literature and even in the Breast Imaging Reporting and Data System recommendations^(5)^. Other indications that are more well-established in the literature^(6-8)^, such as screening for high-risk patients, locoregional staging, and assessment of the response to neoadjuvant treatment, account for a lower proportion of the MRI examinations requested.

Both of the studies cited above were carried out at referral centers for cancer, which suggests that their findings cannot be extrapolated to all facilities in Brazil. However, the factors related to those findings merit further discussion. Unfortunately, the high cost and limited availability of breast MRI are still important limiting factors for the dissemination of the method in the country. Although breast MRI is already a well-established method, there are few facilities in Brazil where this examination is performed by trained professionals, which also impedes the dissemination of the method and even results in a lack of confidence on the part of the requesting physicians. There are even fewer facilities that perform imaging-guided biopsy of suspicious lesions that are detected on MRI and have not been identified by other methods (mammography and ultrasound), which makes the management of such lesions more difficult in some cases.

Updating the data from our previous study^(2)^, we observed that, among the 1,837 breast MRI examinations performed at our center between February 2019 and February 2020 (prior to the current pandemic), the main indications were as follows: diagnostic uncertainty after mammography or ultrasound, in 490 cases (26.7%); surveillance after breast cancer treatment, in 423 cases (23.0%); staging, in 255 cases (13.9%); screening of high-risk patients, in 226 cases (12.3%); and assessment of the response to neoadjuvant chemotherapy, in 145 cases (7.9%). These data suggest a change in the profile of the tests requested over time, with a reduction in indications due to equivocal findings and a proportional increase in other indications. Therefore, we believe that better training and supervision at facilities that perform breast MRI, as well as greater dissemination and discussion of the most appropriate indications for the method among requesting physicians, can favor the optimized use of this resource, making it more cost-effective, in Brazil.
